# Plasma levels of apelin are reduced in patients with liver fibrosis and cirrhosis but are not correlated with circulating levels of bone morphogenetic protein 9 and 10

**DOI:** 10.1016/j.peptides.2020.170440

**Published:** 2021-02

**Authors:** Nicola E. Owen, Duuamene Nyimanu, Rhoda E. Kuc, Paul D. Upton, Nicholas W. Morrell, Graeme J. Alexander, Janet J. Maguire, Anthony P. Davenport

**Affiliations:** a Experimental Medicine and Immunotherapeutics, University of Cambridge, Level 6, Centre for Clinical Investigation, Box 110, Addenbrooke’s Hospital, Cambridge, CB2 0QQ, UK; b Department of Medicine, University of Cambridge, Addenbrooke’s Hospital, Cambridge, CB2 0QQ, UK; c Institute for Liver and Digestive Health, Upper 3rd Floor, Division of Medicine, University College London, Royal Free Campus, Rowland Hill Street, London, NW3 2PF, UK

**Keywords:** Apelin, Apelin receptor, Liver cirrhosis, Fibrosis, BMP9, BMP10, Liver disease

## Abstract

**Background:**

The peptide apelin is expressed in human healthy livers and is implicated in the development of hepatic fibrosis and cirrhosis. Mutations in the bone morphogenetic protein receptor type II (BMPR-II) result in reduced plasma levels of apelin in patients with heritable pulmonary arterial hypertension. Ligands for BMPR-II include bone morphogenetic protein 9 (BMP9), highly expressed in liver, and BMP10, expressed in heart and to a lesser extent liver. However, it is not known whether reductions in BMP9 and/or BMP10, with associated reduction in BMPR-II signalling, correlate with altered levels of apelin in patients with liver fibrosis and cirrhosis.

**Methods:**

Plasma from patients with liver fibrosis (n = 14), cirrhosis (n = 56), and healthy controls (n = 25) was solid-phase extracted using a method optimised for recovery of apelin, which was measured by ELISA.

**Results:**

Plasma apelin was significantly reduced in liver fibrosis (8.3 ± 1.2 pg/ml) and cirrhosis (6.5 ± 0.6 pg/ml) patients compared with controls (15.4 ± 2.0 pg/ml). There was no obvious relationship between apelin and BMP 9 or BMP10 previously measured in these patients. Within the cirrhotic group, there was no significant correlation between apelin levels and disease severity scores, age, sex, or treatment with β-blockers.

**Conclusions:**

Apelin was significantly reduced in plasma of patients with both early (fibrosis) and late-stage (cirrhosis) liver disease. Fibrosis is more easily reversible and may represent a potential target for new therapeutic interventions. However, it remains unclear whether apelin signalling is detrimental in liver disease or is beneficial and therefore, whether an apelin antagonist or agonist have clinical use.

## Highlights

• Apelin has been implicated in hepatic fibrosis and cirrhosis suggesting that plasma levels of this peptide might be a biomarker of liver disease.
• Previous studies of fibrosis and cirrhosis patients reported variable plasma concentrations of apelin ranging from 200 pg/ml to 10,000 pg/ml.
• Using solid-phase extraction, we report decreased plasma levels of apelin in fibrosis and cirrhosis patients compared to healthy volunteers.
• Decreased circulating apelin may be a biomarker of liver fibrosis that occurs in early-stage liver disease.
• Fibrosis, unlike cirrhosis, is easily reversible and may represent a target for new therapeutic interventions.

## 1 Introduction

The peptide apelin-36 was identified in 1998 from bovine stomach as the endogenous ligand for an orphan Class A, G protein-coupled receptor encoded by the angiotensin-like-receptor 1 (AGTRL1, also known as APJ) gene. A shorter isoform, [Pyr^1^]-apelin-13, was subsequently found to be the most abundant in human plasma [1] and cardiovascular system [2]. In humans, apelin has emerged as having an important role in normal cardiovascular physiology, increasing cardiac contractility and acting in an autocrine/paracrine manner to cause vasodilatation by binding to apelin receptors expressed on endothelial cells to release vasodilators [3,4]. In the periphery, apelin is highly expressed in endothelial cells [5], that line blood vessels, including the arterial endothelial cells and hepatic arterioles in the portal tract from healthy livers [6]. Apelin protein expression, measured by western blotting, was increased in the cirrhotic compared to healthy liver, localising to periportal capillary endothelial cells and proliferated arterial capillaries in the fibrotic septa [6]. The consequences of the increased endogenous ligand may be compounded by alterations in receptor expression. For example, the apelin receptor was reportedly upregulated by hypoxia and pro-inflammatory factors in human stellate cells and hepatocytes [7,8]. Indeed, apelin has recently been implicated in the development of hepatic fibrosis and cirrhosis [9,10], suggesting that plasma levels of the peptide might be used as a biomarker of disease.

A limited number of studies have measured apelin levels in the plasma of patients with liver disease, including cirrhosis, but correlations between disease state and peptide concentration have been variable. Two studies [11,12] reported *increased* circulating levels of apelin in patients with cirrhosis, with one of the studies reporting the highest levels in patients with the most severe liver disease [12]. In contrast, a third study by Kalafateli et al. [13] found a significant *reduction* in circulating apelin levels in those patients with the most severe liver disease (alcohol-related cirrhosis). A study examining a group of patients with non-alcoholic fatty liver disease (NAFLD) with early fibrosis reported higher levels of apelin-36 in sera within the disease group [14]. A further study [15] in patients with NAFLD also demonstrated higher levels of apelin in patients within the disease group, but on multivariate analysis, levels were not increased in non-diabetic and normotensive male subjects with NAFLD compared to controls. Despite four of the five studies using an ELISA from the same manufacturer to measure immunoreactive apelin in plasma or sera, the concentration of apelin reported was variable, ranging from 200 pg/ml [15] to 10 ng/ml [13].

Bone morphogenetic protein 9 and 10 (BMP9, BMP10) are circulating cytokines belonging to transforming growth factor-β (TGF-β) superfamily that are paracrine modulators of endothelial cell function [16,17]. Intriguingly, BMP9 is highly expressed in the liver [18,19], while BMP10 is expressed in the heart and, to a lesser extent, liver [20,21]. Both cytokines bind and activate receptor complexes on endothelial cells comprising the bone morphogenetic protein receptor type II (BMPR-II) and the type I receptor, ALK1 [17]. Recently, it has been established that mutations in BMPR-II, that prevent signalling by this receptor, resulted in reduced levels of apelin in both plasma and endothelial cells from patients with heritable pulmonary arterial hypertension (PAH) [22]. However, it is not known whether reductions in BMP9 and/or BMP10, which also result in reduced BMPR-II signalling, would also alter levels of apelin. Cirrhosis is caused by a number of conditions, including alcohol-related and non-alcoholic liver disease, autoimmune hepatitis, hepatitis B and C, primary biliary and sclerosing cholangitis. However, the pathophysiological outcome of these is the same, namely a late stage of scarring of the liver. We have previously discovered that levels of BMP9 and 10 are significantly reduced in the plasma of patients with cirrhosis compared with healthy controls [23]. This provided the opportunity to compare two groups differing in plasma levels of BMP9 and 10. We hypothesised that apelin levels would also be downregulated in cirrhosis. We have previously developed a solid-phase extraction method to minimise variation for the quantification of apelin immunoreactivity in plasma using ELISA. The assay was optimised for the recovery of the peptide, with the identity of the apelin immunoreactivity detected in the ELISA confirmed using liquid chromatography and mass spectrometry. We showed that this method was reproducible [24]. Owing to the variability in the reported levels of apelin peptides in liver disease patients [25,26], we have used this method to extract apelin from plasma samples from the same cohort in which BMP9 and BMP10 were measured and quantified apelin levels by ELISA. We subsequently assessed the data for correlations between apelin plasma levels with those of BMP9 and 10 in the same cohort, as well as patient demographics.

## 2 Methods

### 2.1 Sample collection

Venous blood was collected from patients with fibrosis (n = 14), cirrhosis (n = 56), and controls (n = 25) into EDTA tubes to inhibit apelin metabolising enzymes, mainly metalloproteinases [24]. Blood samples were immediately centrifuged at 3000×*g*, 4 °C, for 10 min and plasma stored at −70 °C. Liver patient samples were collected with informed written consent (IRAS Project 6054, IRAS Project 83963) and with ethical approval from the Health Research Authority's Research Ethics Committee (REC references: 09/H0305/68 and 11/NE/0356, respectively). Healthy control blood samples were from the National Health Service Blood Transfusion as part of the Non-Clinical Issue Service. The patients with liver disease in this cohort had well characterised liver disease as previously described in Owen et al. [23]. This was defined as having had a liver biopsy, revealing fibrosis or cirrhosis, within one year of the study blood sample being taken or evidence of cirrhosis with portal hypertension as defined by the radiological presence of ascites, splenomegaly, or varices. The stage of liver disease was based on standardised histological assessment. Some patients with cirrhosis had not undergone a liver biopsy but were diagnosed based on the radiological findings as described above.

### 2.2 Solid-phase extraction

To reduce interference from proteins, plasma samples underwent an initial peptide extraction step, as previously described [24]. Precision and accuracy measurements demonstrated that the extraction method was robust and reproducible (see Nyimanu et al., 2019 [24], for detailed method and results). With each extraction, plasma samples were spiked with a known concentration of [Pyr^1^]apelin-13 (50 ng/ml) to check the efficiency of peptide recovery.

Plasma aliquots (500 μl) were transferred to pre-chilled 96-well protein Lobind plates (Eppendorf, Stevenage, UK) containing 2 M HCl (125 μl) and vortexed. Plates were then spun at 4000 rpm, 4 °C, for 10 min, and supernatants transferred to 96-well Oasis Primed HLB μ-Elution plates (Waters, Wilmslow, UK). Samples were solid-phase extracted using the Waters Positive Pressure 96 Processor (Wilmslow, UK) and washed with 200 μl of 5 % methanol:1 % acetic acid in water. The samples were subsequently eluted with 60 % methanol:30 % H_2_O:10 % acetic acid (50 μl) into 96-well protein Lobind plates with the elution step repeated four times, followed by centrifugation at 1200 rpm, 4 °C, for 2 min. Samples were dried down (Waters SPE Dry 96 System (Wilmslow, UK)) under nitrogen and stored at −20 °C until required.

### 2.3 Apelin ELISA

Samples were analysed by ELISA (Phoenix Pharmaceuticals, California, USA) according to the manufacturer’s protocol. Cross-reactivity is reported as 100 % for the apelin isoforms apelin-12, apelin-13, apelin-17, and apelin-36. For additional robustness, an 8-point standards curve was used (0.0001–100 ng/ml) to permit more precise interpolation of the plasma sample concentrations from the linear range of the standards curve. Extracted samples were resuspended in the buffer solution (110 μL), and 50 μl of samples and apelin-12 standards were transferred to the ELISA plate. ELISA plates were incubated for 2 h at room temperature with orbital shaking (300–400 rpm). Samples were then repeatedly washed and blot dried (4 times) before adding streptavidin-horseradish peroxidase and incubating for 1 h at room temperature with shaking. Plates were further washed and blot dried, as previously described. 3,3’,5,5'-Tetramethylbenzidine substrate solution (100 μl) was added, and the plates incubated for 1 h at room temperature with orbital shaking. The reaction was stopped by the addition of 2 N HCl (100 μl). Absorbance was read at 450 nm (BioTek Synergy HT Reader, Swindon, UK). The standards curve was constructed using GraphPad Prism 8 for Windows (GraphPad Software, La Jolla, CA, USA) and fitted using a four-parameter logistics equation. The concentration of apelin immunoreactivity in plasma samples was determined by interpolation from the standards curve. We have investigated whether the apelin plasma levels obtained in this study show any correlation with our previously published plasma levels of BMP9 and BMP10 in the same individuals [23].

### 2.4 Statistical analysis

All data sets showed evidence of non-normality (D’Agostino & Pearson omnibus normality test) and were subsequently analysed using Kruskal Wallis or Mann-Whitney tests, as appropriate. Correlation analysis was made using the Spearman non-parametric correlation. Multiple linear regression was also performed on all variables for evidence of independent association with plasma apelin. *p*-values <0.05 were considered statistically significant. Statistical analyses were performed using GraphPad Prism 8.4.3 for Windows.

## 3 Results

### 3.1 Patient demographics

The values for the median age were controls (46.0 years, interquartile range 35.5–56.5), fibrosis (56.5 interquartile range, 45.8–72.25 years), and cirrhosis (58.0 interquartile range, 49.3–66.0 years). The demographics of the patient groups are shown in Table 1. Compared to the fibrotic group, blood levels of bilirubin (11.29 ± 5.9 vs 50.79 ± 19.0, *p* < 0.0001) and prothrombin time (11.94±0.9 vs 14.60±0.6, *p* < 0.0001) were significantly increased, while blood sodium (139.79±2.0 vs 138.29±0.8, *p* < 0.001), albumin (40.07±3.0 vs 34.07±2.3, *p* < 0.0001) and platelet count (218.64±51.0 vs 114.71±12.7, *p* < 0.0001) were significantly decreased in the cirrhotic group. The patients in this cohort had well-characterised liver disease. This was defined as having a liver biopsy within one year of the blood sample being taken. The stage of liver disease was based generally on histological assessment. In a small number (n=10) of cirrhotic patients, who had not had a liver biopsy, the staging was based on the radiological presence of ascites, splenomegaly, or varices on endoscopy. The control samples were from healthy active blood donors who fulfilled the criteria for blood donation and screened for viral hepatitis, including hepatitis B, C, D, and E. Both the age and sex of the donors were obtained.

**Table 1:** Demographics of healthy controls, fibrotic and cirrhotic patients.

	Healthy controls n=25	Fibrosis n=14	Cirrhosis n=56
***Age, years***	44.9±2.7	59.8±3.9	56.2±1.4
***Gender (%)***			
Male (%)	11 (44)	8 (57)	29 (52)
Female (%)	14 (56)	6 (43)	27 (48)

***Aetiology (%)***			
AIH		8 (57)	5 (9)
A1AT			1 (1.8)
AIH/A1AT			1 (1.8)
ArLD/A1AT			1 (1.8)
ArLD			19 (34)
HBV			2 (3.5)
HCV		1 (7)	9 (16)
HBV/HAFLD			1 (1.8)
HCV/ArLD			3 (5.4)
PBC		1 (7)	1 (1.8)
PBC/AIH		1 (7)	4 (7)
PSC		2 (14)	
NAFLD		1 (7)	5 (9)
NAFLD/ArLD			2 (3.5)
Sarcoid			2 (3.5)

***Blood Markers***			
Bilirubin (μmol/l)		11.29±5.9	50.79±19.0***
Albumin (g/l)		40.07±3.0	34.07±2.3***
Sodium (mmol/l)		139.79±2.0	138.29±0.8**
Creatinine (μmol/l)		71.29±12.3	68.14±5.6
Platelet count (10*9/l)		218.64±51.0	114.71±12.7***
Prothrombin time (sec)		11.94±0.9	14.60±0.6***

***Severity Scores***			
INR, score (%)		0.9 (7)	1 (21.4)
		1 (43)	1.1 (14.3)
		1.1 (50)	1.2 (8.9)
			1.3 (12.5)
			1.4 (12.5)
			1.5 (10.7)
			1.6 (7.1)
			1.7 (3.6)
			2 (5.4)
			2.2 (1.8)
			2.7 (1.8)
Ascites, score (%)			1 (58.9)
			2 (14.3)
			3 (26.8)
HE, score (%)			0 (82)
			2 (9)
			3 (9)

Data are mean±SEM. **P<0.001, *** *P<*0.0001 cirrhotic values significantly different from fibrosis. A1AT - alpha 1 anti-trypsin, AIH - autoimmune hepatitis, ArLD - alcohol-related liver disease, HBV - hepatitis B virus, HCV - hepatitis C virus, INR - international normalised ratio, NAFLD - non-alcoholic liver disease, PBC - primary biliary cholangitis, PSC - primary sclerosing cholangitis. The Child-Pugh Score for ascites; 1 = absence of ascites, 2 = ascites mild or supressed with medication, 3 = ascites moderate to severe (or refractory). Hepatic encephalopathy (HE) was scored using West Haven criteria; 0 = absence of HE symptoms, 1 = changes in behaviour with minimal change in level of consciousness, 2 = disorientation, drowsiness, inappropriate behaviour, 3 = marked confusion, incoherent speech, sleeping but rousable to vocal stimuli.

### 3.2 Measurement of apelin immunoreactivity in plasma

The inter-assay coefficient of variation from three separate assays at three different concentrations of apelin from low to high was 4.96 % (1.6 pg/ml, 1 pmol/l), 2.2 % (16 pg/ml, 10 pmol/l), 5.30 %, (1E-10 M, 160 pmol/l) and 5.40 % (1E-8 M, 1600 pmol/l). The inter-assay coefficient of variation within a plate was 2.86 %. These fall well within the generally accepted values of <15 % for inter-assay and <10 % for intra-assay coefficients of variation. Recovery of [Pyr^1^]-apelin 13 in plasma was high at 77 %. The sensitivity of the assay was calculated to be 23 pg/ml. All values were within the optical density range encompassed by the standards curve except for three samples that were beyond the range and excluded from the analysis.

**Fig. 1:**
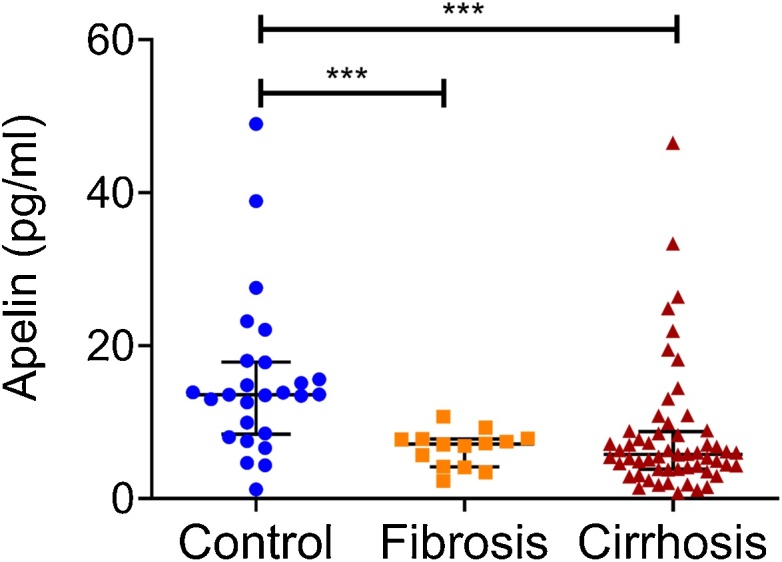
Apelin plasma levels compared in controls (n = 25) and patients with fibrosis (n = 14) or cirrhosis (n = 56). Mann Whitney test, ***p < 0.0001 indiates significant difference from control group. Horizontal bars show the median and interquartile range.

**Fig. 2:**
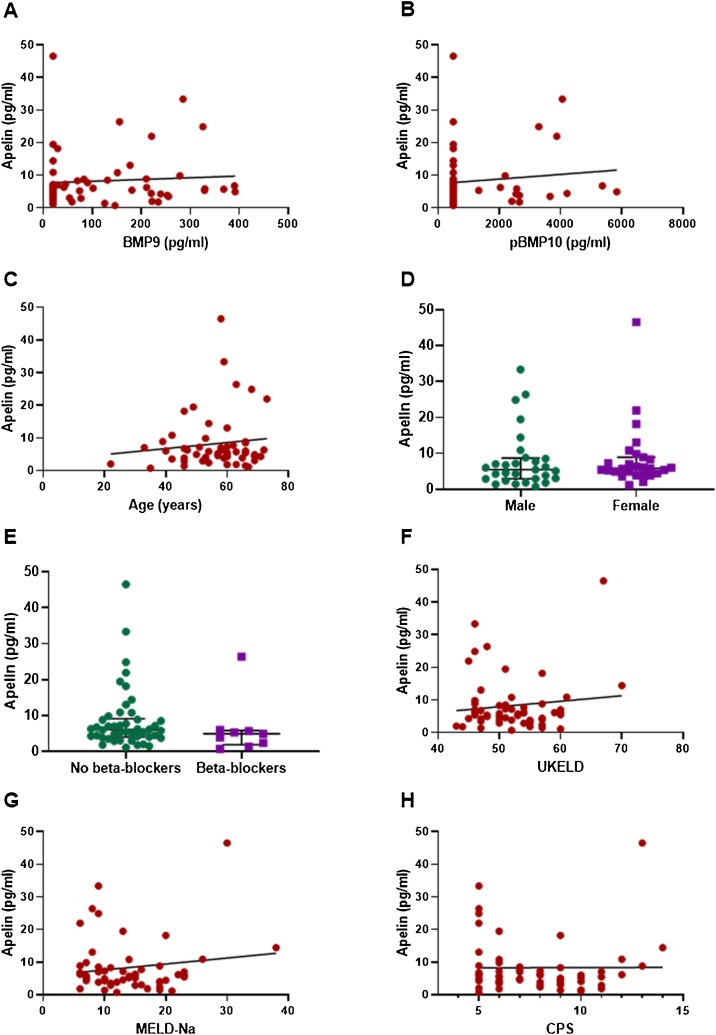
Circulating apelin levels in patients with cirrhosis do not correlate with circulating BMP9 (A), pBMP10 levels (B), age (C), and liver disease severity scores (F, G, and H). In patients with cirrhosis, there are no differences in circulating apelin levels between males and females (D) and those on or off beta-blockers (E). Fig. 2A, B, C, F, G, and H: Spearman non-parametric correlation. Fig. 2D and H: Mann Whitney Test. Error bars show the median and interquartile range. Abbreviations: CPS – Child Pugh Score, MELD-Na – Model for End-stage Liver Disease incorporating sodium values, UKELD – United Kingdom model for End-stage Liver Disease. Individual levels of BMP9 and BMP10 (as pBMP10) have previously been published by us [23].

There was a significant reduction in apelin levels in patients with both fibrosis (6.5 ± 0.6 pg/ml) and cirrhosis (8.3 ± 1.2 pg/ml) compared to healthy controls (15.4 ± 2.0 pg/ml) (Fig. 1). As previously reported, unlike apelin, levels of BMP9 and pBMP10 are not significantly reduced in patients with fibrosis compared to healthy controls (BMP9, 218.1 ± 9.5 vs 315.6 ± 24.7 pg/ml, *p*>0.05; pBMP10, 3479.6 ± 298.3 vs 2909.2 ± 458.2 pg/ml, *p* > 0.05). However, both peptides, like apelin, are significantly reduced in patients with cirrhosis (BMP9, 218.1±9.5 vs 124.9±15.7 pg/ml, *p<*0.002; pBMP10, 3479.6 ± 298.3 vs 1236.1 ± 184.6 pg/ml, *p* < 0.0001). Within the cirrhotic group, there was no correlation of plasma apelin with levels of either BMP9 or pBMP10, age or sex (Fig. 2A–D). There was no significant correlation between apelin concentration and whether patients were untreated or treated with β-blockers, the current predominant standard of care for significant portal hypertension with varices (*r* = 0.02, *p* > 0.05). There was no difference in mean plasma concentrations of apelin between these treatment groups (Fig. 2E). However, there were only nine patients on β-blockers compared to forty five that were untreated and therefore, it is not possible to determine if β-blockers had any effect. Within the cirrhotic group, there was no significant correlation between levels of apelin and measures of disease severity scores UKELD, MELD-Na (widely used for prognostication for liver transplants), and Child-Pugh Score (a measure of disease prognosis for one- and five-year survival) (Fig. 2F–H).

### 3.3 Multiple linear regression analysis

In multiple linear regression analysis, BMP9 (*t* = 0.23, *p* = 0.82), pBMP10 (*t* = 0.75, *p* = 0.45), age (*t* = 0.40, *p* = 0.69) and sex (*t* = 0.84, *p* = 0.40) were not independently associated with plasma apelin concentration in patients (Table 2). Circulating apelin concentration in patients was not independently associated with markers of liver disease including bilirubin (*t* = 0.015, *p* = 0.98), albumin (*t* = 1.81, *p* = 0.07), sodium (*t* = 0.91, *p* = 0.36), platelet count (*t* = 1.03, *p* = 0.31), creatinine (*t* = 0.4, *p* = 0.69) or prothrombin time (*t* = 0.47, *p* = 0.64) (Table 3).

**Table 2:** Multiple linear regression analysis showing BMP9, BMP10, age or sex were not independently assoaciate with plasma concentrations of [Pyr^1^]apelin-13.

	Beta coefficient	Standard error	*t* statistic	*p* value
Log BMP9	−0.27	0.12	0.23	0.82
Log pBMP10	0.11	0.15	0.75	0.45
Age	0.001	0.003	0.40	0.69
Sex	−0.07	0.08	0.84	0.40

Abbreviations: BMP9 – bone morphogenic protein 9, BMP10 – bone morphogenic protein 10.

**Table 3:** Multiple linear regression analysis did not show any association between blood test data and plasma apelin.

	Beta coefficient	Standard error	*t* statistic	*p* value
Bilirubin	1.2e-5	7.9e-4	0.015	0.98
Albumin	0.015	0.008	1.81	0.07
Sodium	−0.01	0.013	0.91	0.36
Platelet count (10*8/l)	7.9e-4	7.7e-4	1.03	0.31
Prothrombin time	−0.03	0.06	0.47	0.64
Creatinine	9.2e-4	0.003	0.4	0.69
INR	0.62	0.81	0.76	0.45

INR - international normalised ratio.

## 4 Discussion

In the volunteer controls, mean levels of apelin (uncorrected for recovery, which was 77 %) was 15.4 ± 2.0 pg/ml, which is equivalent to ∼10 pmol/l. This is in line with the plasma concentration of other peptides thought to be locally released from the endothelium, such as endothelin-1, 5.7 pmol/l [27], VIP, 30 pmol/l and neuropeptide FF, 2.7 pmol/l [28], that were also subjected to solid-phase extraction before measurement in an immunological assay. These levels are consistent with apelin being released primarily from endothelial cells and acting in an autocrine/paracrine manner to cause vasodilatation in humans. Previous studies have reported a wide range of apelin levels from 2 pg/ml [29] to 118,000 pg/ml [30]. Our results, as might be expected following solid-phase extraction, are towards the lower end of the scale. Where higher levels have been reported without prior extraction, this may be the result of nonspecific binding and interference by proteins such as albumin and haemoglobin. The method that we used was optimised for good recovery of apelin standards from spiked plasma samples. We also previously confirmed that the analyte being measured is [Pyr^1^]-apelin-13, the most abundant isoform identified in plasma [2], using mass spectrometry [24].

Compared to healthy controls (15.4 ± 2.0 pg/ml), apelin levels were reduced by about 50 % in patients with both earlier-stage (fibrosis) (6.5 ± 0.6 pg/ml) and later-stage liver disease (cirrhosis) (8.3 ± 1.2 pg/ml), indicating that changes in plasma apelin levels may be a useful biomarker for liver disease. Our hypothesis was that changes in plasma apelin levels would positively correlate with the known reduction of BMP9 and BMP10 in cirrhosis. The potential mechanism being a loss of signalling through the BMPR-II receptor, expressed on endothelial cells, leading to a reduction in apelin release, similar to that reported for patients with PAH [22]. However, we have found that the decrease in plasma apelin occurs at an earlier stage of liver disease than for BMP9 and BMP10 suggesting that if BMPR-II signalling is impaired this is not mediated via loss of these receptor ligands. BMPR-II is activated by BMP2, BMP4 and BMP7 in addition to BMP9 and BMP10, and although activation of the receptor by some of these ligands was suggested to inhibit apelin expression, especially in the context of angiogenesis, the opposite effect was observed for BMP2 which increased apelin expression downstream of BMPR-II [31,32]. Therefore, the lack of correlation between plasma concentration of apelin and BMP9 or BMP10 observed in these patients may partly be explained by the actions of other BMPR-II ligands, such as BMP4, 2 or 7.

The precise role of apelin in the pathophysiology of liver disease in general and fibrosis, in particular, is unclear [9,25,26]. Injury to hepatic stellate cells, such as in patients in this study, is key to secreting fibrogenic factors that cause fibrocytes, fibroblasts, and bone marrow-derived myofibroblasts to produce collagen and fibrosis. Hepatic stellate cells express apelin receptors at low levels in healthy control livers, but these become more prominent in sinusoids and on portal fibroblasts in fibrotic septa from cirrhotic liver [33] and therefore are potentially able to respond to apelin released from endothelial cells, locally or circulating in the plasma. There is an emerging consensus that apelin receptor density is upregulated in liver cirrhosis [9]. Apelin has been proposed as a pro-fibrotic agent [34], mediating the induction of profibrogenic genes in human hepatic stellate cells in vitro [7]. In a rat model of CCl_4_-induced fibrosis, an apelin ligand, F13A-apelin (where the terminal phenylalanine has been substituted for a structurally similar amino acid, alanine) was found to decrease fibrosis by 25 % compared to vehicle‐treated cirrhotic rats [11]. Similar results were also obtained by [35] in this by infusion of F13A-apelin. These results are difficult to interpret as the consensus is that in humans, F13A-apelin acts as an apelin receptor agonist [36] activating apelin signalling, rather than an antagonist, blocking this pathway. Surprisingly, in all other organ systems studied to date (renal, cardiac, and pulmonary), the apelin signalling pathway is beneficial in reducing fibrosis [37].

In contrast, beneficial effects of apelin in the liver of an animal model have been reported using apelin linked to a fusion protein to increase plasma half-life (∼33 h), which attenuates lipopolysaccharide-induced liver injury in mice including fibrosis [38]. Our results suggest that apelin signalling pathway is reduced in fibrosis, an early stage in the development of liver disease. Crucially it is considered that fibrosis can be more easily reversed at this stage, unlike cirrhosis, and intriguingly may represent a potential drug target.

In other models, apelin has been proposed to be protective: in a mouse model of hepatocellular steatosis, the apelin signalling pathway was protective in preventing lipid accumulation in hepatocytes [39]. Chronic apelin treatment improved hepatic lipid metabolism in obese and insulin-resistant mice [40]. In mouse primary hepatocytes and liver tissues, apelin ameliorates TNF-alpha-induced reduction of glycogen synthesis [41].

Our results show, using a validated assay system with solid-phase extraction to reduce interference, apelin immunoreactive levels are reduced in patients with fibrosis and cirrhosis. It is unclear whether lowered levels of apelin are beneficial or detrimental in cirrhosis. It is also unclear from experimental studies in animal models whether the apelin signalling pathway should be blocked by the addition of apelin antagonist or whether low levels of the endogenous peptide should be enhanced as in PAH. In animal models of PAH, a G protein biased apelin agonist with reduced potency at the β-arrestin pathway and therefore reduced likelihood of producing receptor desensitisation has been shown to be effective in attenuating the symptoms [42] and has been used in experimental human studies [43]. Conversely, biased apelin ligands have also been shown to be effective in blocking detrimental actions of apelin, for example, in a mouse ectopic xenograft tumour model (glioblastoma) showed a reduction in tumour growth compared to control and improved survival of tumour-bearing mice [44]. A range of pharmacological tools [3,4], have been developed that can be used to more precisely delineate the role of the apelin signalling pathway in the liver. Some of these have been shown to be effective in animal models such as PAH, where apelin levels are reduced [42,45] as well as experimental medicine studies [43] with the prospect of translation into the clinic.

## Grant information

This research was supported by Addenbrooke’s Hospital Hepatology Research Fund (NEO), 10.13039/100010269Wellcome Trust [WT107715/Z/15/Z] (APD), British Heart Foundation Programme grants (RG/13/4/30107 and RG/19/3/34265) (PU and NWM). We thank the Cambridge Biomedical Research Centre Biomedical Resources Grant (University of Cambridge, Cardiovascular Theme, RG64226).

## Author contribution

DN and NEO designed and performed experiments, analysed data, wrote the manuscript, REK and PDU performed experiments, GJA, NWM reviewed the manuscript, JJM, APD provided experimental design, data analysis, wrote the manuscript, supervision of the project and funding.

## CRediT authorship contribution statement

**Nicola E. Owen:** Conceptualization, Formal analysis, Investigation, Writing - original draft, Writing - review & editing. **Duuamene Nyimanu:** Conceptualization, Formal analysis, Investigation, Methodology, Writing - original draft, Writing - review & editing. **Rhoda E. Kuc:** Investigation. **Paul D. Upton:** Investigation. **Nicholas W. Morrell:** Writing - review & editing. **Graeme J. Alexander:** Writing - review & editing. **Janet J. Maguire:** Conceptualization, Formal analysis, Funding acquisition, Supervision, Writing - original draft, Writing - review & editing. **Anthony P. Davenport:** Conceptualization, Formal analysis, Funding acquisition, Supervision, Writing - original draft, Writing - review & editing.

## Declaration of Competing Interest

The authors report no declarations of interest.
